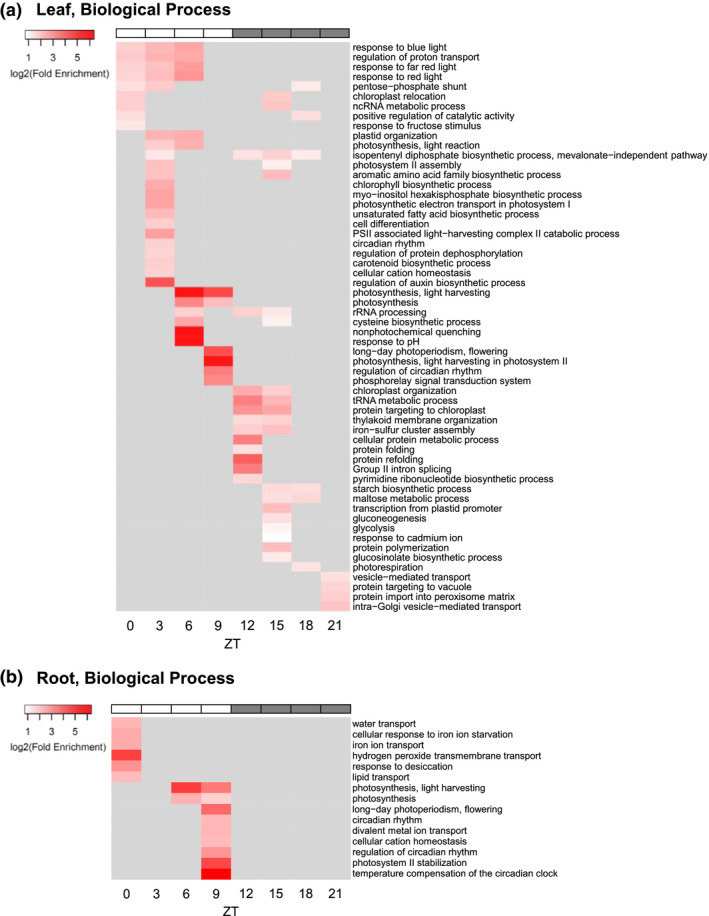# Corrigendum

**DOI:** 10.1002/pld3.327

**Published:** 2021-05-07

In the article by Matsuda et al., the GO terms were labeled in wrong order in Figure 4A.

In the section 2.6 LC‐MS/MS analysis, the authors indicated a wrong gradient program. The correct program is as follows: “The gradient program was isocratic at 10% D, Initial; linear at 0%–85% D, 0–15 min; isocratic at 100% D, 15–16 min; and isocratic at 10% D, 16–20.5 min.”

In the section 2.2 Plant materials and growth conditions, the authors indicated a wrong pH for the medium. pH of a mineral nutrient medium was 5.5 instead of 6.0.

The authors apologize for the errors from their published article.